# TrigNER: automatically optimized biomedical event trigger recognition on scientific documents

**DOI:** 10.1186/1751-0473-9-1

**Published:** 2014-01-08

**Authors:** David Campos, Quoc-Chinh Bui, Sérgio Matos, José Luís Oliveira

**Affiliations:** 1 IEETA/DETI, University of Aveiro, 3810-193, Aveiro, Portugal; 2 Department of Medical Informatics, Erasmus Medical Centre Rotterdam, Rotterdam, Netherlands

## Abstract

**Background:**

Cellular events play a central role in the understanding of biological processes and functions, providing insight on both physiological and pathogenesis mechanisms. Automatic extraction of mentions of such events from the literature represents an important contribution to the progress of the biomedical domain, allowing faster updating of existing knowledge. The identification of trigger words indicating an event is a very important step in the event extraction pipeline, since the following task(s) rely on its output. This step presents various complex and unsolved challenges, namely the selection of informative features, the representation of the textual context, and the selection of a specific event type for a trigger word given this context.

**Results:**

We propose TrigNER, a machine learning-based solution for biomedical event trigger recognition, which takes advantage of Conditional Random Fields (CRFs) with a high-end feature set, including linguistic-based, orthographic, morphological, local context and dependency parsing features. Additionally, a completely configurable algorithm is used to automatically optimize the feature set and training parameters for each event type. Thus, it automatically selects the features that have a positive contribution and automatically optimizes the CRF model order, n-grams sizes, vertex information and maximum hops for dependency parsing features. The final output consists of various CRF models, each one optimized to the linguistic characteristics of each event type.

**Conclusions:**

TrigNER was tested in the BioNLP 2009 shared task corpus, achieving a total F-measure of 62.7 and outperforming existing solutions on various event trigger types, namely gene expression, transcription, protein catabolism, phosphorylation and binding. The proposed solution allows researchers to easily apply complex and optimized techniques in the recognition of biomedical event triggers, making its application a simple routine task. We believe this work is an important contribution to the biomedical text mining community, contributing to improved and faster event recognition on scientific articles, and consequent hypothesis generation and knowledge discovery. This solution is freely available as open source at http://bioinformatics.ua.pt/trigner.

## Background

A growing amount of biomedical data is continuously being produced, resulting largely from the widespread application of high-throughput techniques, such as gene and protein analysis. This growth is accompanied by a corresponding increase of textual information, in the form of articles, books, and technical reports. In order to organize and manage these data, several manual curation efforts have been set up to identify entities (e.g., genes and proteins), their interactions (e.g., protein-protein) and events (e.g., gene transcription and regulation). The extracted information is then stored in structured knowledge resources, such as Gene Ontology [1] and Swiss-Prot [2]. However, manual curation of large quantities of data is a very demanding and expensive task, being difficult to keep these databases up-to-date. These factors have naturally led to increasing interest in the application of text mining (TM) systems to help perform those tasks.

Biomolecular events such as gene transcription, protein binding or cell cycle regulation, play a key role in the interpretation of biological processes and cellular functions. For instance, a given protein may regulate the expression of a gene, whose products are in turn involved in some biological process. These events, as well as their biological significance and impact, are usually described in the scientific literature, and building up the complex chains of events that compose a biological network is a very demanding and time-consuming task. Additionally, the yielded knowledge can also be used by the pharmaceutical industry for both drug discovery and design, as the identification of proteins involved in key events might result in the subsequent uncovering of new drug targets. Thus, automatic event extraction from text constitutes an important contribution, in order to help find hidden biological relationships and allow faster updating of existing knowledge.

**Figure 1:**
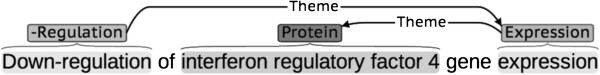
Textual representation of a complex biomedical event.

Textual representation of biological events typically occurs as a relation between a word indicating the event, which we call the trigger, and one or more arguments, which may be a biomedical concept or another event. For instance, Figure 1 contains two different biological events: 1) Gene Expression between the trigger word “expression” and the protein “interferon regulatory factor 4″; and 2) Negative Regulation between the trigger “Down-regulation” and “expression”, representing event 1.

The development of automatic solutions to extract biological events from scientific documents has been greatly promoted by the BioNLP shared tasks [3,4], aimed at the recognition of events particularly focused on genes and proteins. More recently, the extraction of events focused on infectious diseases, bacteria and cancer genetics were also targeted. In general, the proposed approaches to event extraction consist of two subsequent sub-tasks:

● Trigger recognition: aimed at identifying the chunk of text that triggers the event and serves as a predicate;

● Argument recognition: aimed at identifying the entity and/or event that take part in the event.

Trigger recognition is the first and crucial task of event recognition, since the following task(s) completely rely on its output. This was clearly shown by Björne et al. [5], who stated a drop of more than 20 points in performance between using predicted and gold standard triggers. However, trigger recognition presents various complex and unsolved challenges, namely:

● The same chunk of text may be a trigger word or not depending on the textual context;

● The same chunk of text may be a trigger of two or more event types;

● Triggers of different event types have different linguistic characteristics;

● Large amount and variety of event types.

Approaches to perform event trigger recognition can be categorized as being based on rules, dictionary matching and machine learning. Rule-based approaches apply a set of manually or automatically generated linguistic rules to extract trigger words. For instance, Cassillas et al. [6] identified the most common trigger-based patterns from training data using lemmas, such as “phosphorylat* + of + PROTEIN”, where “phosphorylat*” represents the trigger. Regarding dictionary-based solutions, developers need to collect trigger words for each event type, in order to build a focused knowledge resource, i.e., dictionary. In the end, the words in the dictionary are matched with the text and accepted as triggers for each event type. However, such an approach accepts all trigger words without considering the textual context, possibly producing large amounts of false positives. To minimize this problem, manual linguistic rules can be applied, in order to filter provided triggers and significantly reducing the amount of false positives. For instance, Minh et al. [7] accepts only words that are present in specific contexts and with specific part-of-speech tags, such as “NN/NNS + of + PROTEIN” and “VBN + PROTEIN”. On the other hand, Kilicoglu and Bergler [3,4,8] applied statistical measures based on linguistic features to collect “good” trigger words from training data.

Machine learning (ML) based solutions use statistical models focused on recognizing specific words by applying a feature-based representation of the observed data. Such an approach aims to minimize various problems of rule and dictionary-based solutions, namely regarding context definition. ML-based solutions vary with the used statistical model and extracted features. Support Vector Machines (SVMs) are the most commonly used ML model for this task. For instance, Björne et al. [5,9] apply SVMs with a complex feature set consisting of tokens, dependency parsing tree and external resources to identify event triggers for each input sentence. The problem of multiple trigger types per chunk of text is solved through the application of composite labels. Miwa et al. [6,10] also took advantage of SVMs, but training two different models: one for trigger-protein (TP-T) relations and another for trigger-trigger (TT-T) relations, using the output of the TP-T predictor as an input feature for the TT-T model. Their system employs a complete feature set based on tokens, local context and dependency parsing with shortest paths features. On the other hand, Zhang et al. [7,11] used SVMs with neighborhood hash features to reflect the syntactic structure of the sentences, in combination with token and sentence-based features. Finally, Martinez and Baldwin [12] used SVMs in the perspective of word sense disambiguation (WSD), by defining a list of target words, i.e., triggers. This solution also used features based on tokens, context, dependency parsing and external resources. Besides SVMs, Conditional Random Fields (CRFs) have also been applied, presenting state-of-the-art results on sequence tagging problems. For instance, MacKinlay et al. [13] used CRFs with a feature set based on token, dependency parsing and context definition features. Martinez and Baldwin [12] also applied CRFs using a similar feature set as applied in the WSD approach. Overall, the results presented so far show that SVMs offer better performance, but we believe that CRFs have not yet been properly and deeply explored in the task of trigger event recognition.

ML-based approaches were the most commonly used in previous BioNLP event extraction challenges, followed by dictionary-based systems and rule-based solutions. Regarding performance behavior, ML-based solutions present the best results, followed by dictionary matching approaches. However, current ML-based approaches still present various limitations, namely:

● The problem of a single chunk of text with multiple trigger types is not properly and generally solved;

● Current solutions do not consider the heterogeneous linguistic characteristics of different event types;

● Feature set selection is typically performed manually;

● Availability of open source solutions is limited;

● Existing solutions are not usually configurable and/or extendable, limiting their application in different domains and with different event types.

This article proposes an advanced, open source and high performance machine learning-based approach for event trigger recognition, aimed at minimizing the aforementioned limitations. It takes advantage of a high-end feature set and is focused on automatic optimization per event type. Such a method makes the application of complex trigger recognition techniques a simple routine task, contributing to improved and faster biomedical event recognition. The following section presents the applied techniques, namely the used feature set and the implemented optimization algorithm. Afterwards, a comparison of achieved performance results is performed, discussing the advantages and limitations of the proposed approach. Finally, some concluding remarks are presented.

## Methods

This section presents the applied processing pipeline and supporting data structure, which will serve as support to extract linguistic features and train machine-learning models to automatically recognize triggers.

### Processing pipeline

**Figure 2:**
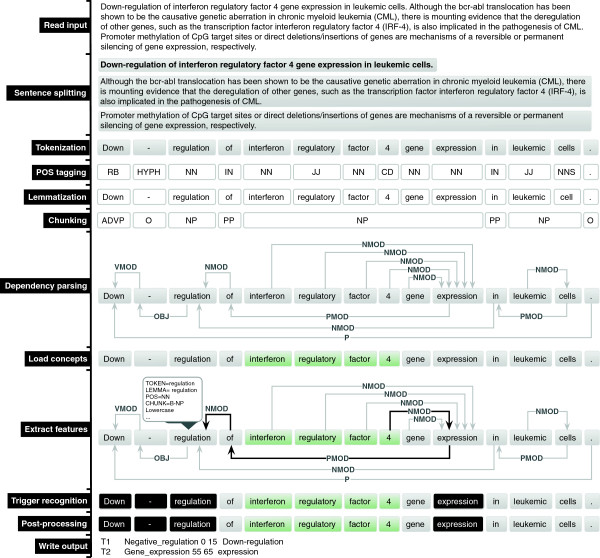
Illustration of the processing pipeline for the sentence “Down-regulation of interferon regulatory factor 4 gene expression in leukemic cells.”, highlighting the output of linguistic parsing, shortest paths, provided concepts and extracted triggers.

Since a trigger recognition solution must be combined with other methods to perform event extraction, such a system must be implemented on top of a modular and flexible architecture, in order to allow easy integration of new modules and respective features. Thus, our solution was developed on top of Neji [14], an open source framework that provides a modular processing pipeline for biomedical concept recognition. Neji integrates various modules optimized for the biomedical domain, such as natural language processing (sentence splitting, tokenization, lemmatization, part-of-speech tagging, chunking and dependency parsing) and concept recognition (dictionaries and machine learning). Popular biomedical input and output formats are also supported. The processing pipeline applied in our system is illustrated on Figure 2, which contains the following general modules and steps:

● Reader: read input data and mark the text regions of interest;

● NLP: perform sentence splitting using LingPipe [15], and tokenization, lemmatization, part-of-speech (POS) tagging, chunking and dependency parsing using a custom version of GDep [16] with optimized tokenization;

● Concept loader: load relevant concepts;

● Dictionary tagger: perform trigger recognition using one or multiple previously built dictionaries;

● Machine learning: perform trigger recognition using one or multiple previously trained models;

● Post-processing: remove false positive trigger names through rule-based approaches;

● Writer: write the output to an external resource.

### Data structure

**Figure 3:**
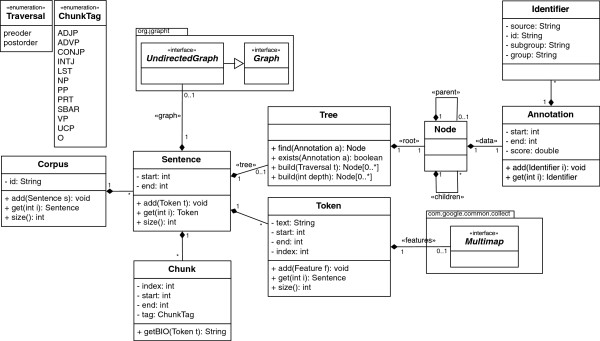
Internal data structure to support a corpus with multiple sentences and associated information, namely tokens, chunks, dependency parsing graph, concept tree and features.

After reading input data in RAW format and performing NLP processing, it is fundamental to store relevant linguistic information in a structured manner, in order to facilitate further processing. Figure 3 illustrates the internal data representation to support all the information associated with a corpus. The core components are sentences and tokens, which provide their relative positions regarding the input text. Chunking output is stored using the target token positions and a label for the corresponding chunk type. Moreover, dependency-parsing output is stored as an undirected graph, where nodes are tokens and edges contain labels to describe each linguistic dependency. Such graph representation allows easy traversing of the various dependencies and extracting paths for any given token. The graph implementation is based on the JGraphT library [17], which contains methods to simplify path and shortest path construction.

The support for other features and/or information associated with each token is provided through a map of keys and values, where a key identifies a type of feature and the value is the feature itself. However, since each feature type may contain multiple values, the mapping is performed between a key and a list of values. This implementation is based on a Multimap from the Guava library [18]. Thus, since lemmas and part-of-speech tags are specific to each token, they are provided as features in the multimap. Moreover, to cope with nested and intersected concept and trigger annotations, it is important to integrate a data structure that suits such characteristics in the best and most automated way. This is achieved through a tree of annotations, which offers various advantages over typical approaches (e.g., list of annotations), such as automatic maintenance of structured annotations and easy identification of ambiguity problems. The extracted and stored information is also illustrated on Figure 2.

### Loading concepts

Since the extraction of biomedical events requires previous annotation of biomedical concepts, we support both loading and automatically identifying those concepts in the texts. If manual annotations are available, they should be provided in A1 format [19] (bottom of figure Figure 2). On the other hand, dictionary or machine learning-based approaches can be applied to perform automatic recognition of such biomedical concepts.

### Dictionary matching

When data containing manual annotations of event triggers are unavailable or scarce, training machine learning models may not be possible. Thus, we also provide the ability to perform trigger recognition using dictionaries. Such functionality is achieved by case-insensitive exact dictionary matching, using deterministic finite automata (DFA) through a custom version of the dk.brics.automaton library [20]. Dictionaries are provided in TSV (tab-separated values) files with two fields: identifier and respective list of names. The responsibility for building such dictionaries is left to the user.

### Machine learning

When ML techniques are applied to trigger recognition, an algorithm must build a feature and statistic-based representation of target names from training data, in order to develop an appropriate response to unseen data. Such methodologies are commonly categorized as being supervised or semi-supervised. Semi-supervised solutions use both annotated and unannotated data, in order to obtain features of the trigger words that are not present in the annotated data. Specifically for this task, the use of unannotated data could contribute to a better abstract learning of triggers. However, the application of such techniques is computationally heavy and could be implemented as an extension to an equivalent supervised solution. Thus, we decided to follow a supervised training approach, through the application of Conditional Random Fields (CRFs) [21]. Such a technique presents various advantages over other methods. Firstly, CRFs avoid the label bias problem [21], a weakness of Maximum Entropy Markov Models (MEMMs). Additionally, the conditional and discriminative nature of CRFs relaxes strong independence assumptions required to learn the parameters of generative models, such as Hidden Markov Models (HMMs) [22]. Finally, Support Vector Machines (SVMs) follow a different approach and have been shown to deliver high-performance results. However, training complex SVM models may take more time.

Conditional Random Fields (CRFs) were first introduced by Lafferty et al. [21]. Assuming that we have an input sequence of observations (represented by *X*), and a state variable that needs to be inferred from the given observations (represented by *Y*), a “CRF is a form of undirected graphical model that defines a single log-linear distribution over label sequences (*Y*) given a particular observation sequence (*X*)” [22]. This layout makes it possible to have efficient algorithms to train models, in order to learn conditional distributions between *Y*_*j*_ and feature functions from the observable data. To accomplish this, it is necessary to determine the probability of a given label sequence *Y*, given *X*. First, the model assigns a numerical weight to each feature, and then those weights are combined to determine the probability of *Y*_*j*_. Such probability is calculated as follows:

$$p\left(y|x,\lambda \right)=\frac{1}{Z\left(x\right)}\exp\left(\sum_{j}\lambda_{j}F_{j}\left(y,x\right)\right),$$

where *λ*_*j*_ is a parameter to be estimated from training data and indicates the informativeness of the respective feature, *Z*(*x*) is a normalization factor and $F_{j}\left(y,x\right)=\sum_{i=1}^{n}f_{j}\left(y_{i-1},y_{i},x,i\right)$, where each *f*_*j*_(*y*_*i*-1_, *y*_*i*_,*x*,*i*) is either a state function.

*s*(*y*_*i*-1_, *y*_*i*_,*x*,*i*) or a transition function *t*(*y*_*i*-1_, *y*_*i*_,*x*,*i*) [22]. When considering higher-order models, each label depends on a specific number of *o*-previous labels. Thus, the probability will consider not only the previous observation and its features, but *o*-previous observations and features, which produces better model dependencies and may provide improved results, depending on the target data and task. However, the training complexity of higher-order models increases exponentially with the pre-defined order *o*.

The support for CRF models is provided through Gimli [23], an open-source biomedical concept recognition tool based on the MALLET framework [24] that provides high-performance results in two well-known corpora: GENETAG [25] and JNLPBA [26]. Gimli implements a comprehensive set of features optimized for the biomedical domain, therefore serving as a good starting point for trigger recognition.

### Feature set

The proposed solution supports a complex and high-end feature set, extracting features based on tokens, sentences, concepts, dependency parsing trees and external resources. On top of those, different strategies to model local context are also provided.

### Token

Token-based features intend to capture specific knowledge regarding to each token, namely linguistic, orthographic and morphological characteristics. The most basic feature is the token text. However, in most cases, morphological variants of words have similar semantic interpretations, which can be considered as equivalent. For this reason, lemmatization is used to group together inflected forms of a word, so that they can be analyzed as a single item. On the other hand, it is also possible to associate each token with a particular grammatical category based on its definition and context, a procedure called part of speech (POS) tagging. Moreover, we also use chunking, dividing the text into syntactically correlated chunks of words (e.g., noun or verb phrases). The BIO encoding format is used to properly indicate the beginning and end of each chunk. For instance, considering two consecutive tokens that constitute a noun phrase chunk, the tag “B-NP” is associated with the first token and the tag “I-NP” with the second one. In the end, each tag is used as a feature of the respective token. Regarding orthographic features, their purpose is to capture token formation characteristics, through three different types of features:

● Capitalization: reflect uppercase and lowercase characteristics, such as “InitUpp” (token starts with uppercase character) and “MixCase” (token has both lowercase and uppercase characters);

● Counting: count the number of uppercase characters and numbers, and provide token length;

● Symbol: reflect the occurrence of symbol characters, such as dots, commas and semicolons.

On the other hand, morphological features reflect common structures and/or sub-sequences of characters among several tokens, identifying similarities between distinct triggers. Three different types of morphological features are considered: suffixes and prefixes, char n-grams and word shape patterns. Particular prefixes and suffixes could be used to distinguish trigger names, such as the 3-character prefix “coe” for the “coexpression” trigger. A char *n*-gram is a subsequence of *n* characters from a given token, which finds common sub-sequences of characters in the middle of tokens. Finally, it is also important to extract the token’s structure, reflecting how letters, digits and symbols are organized in the token. For instance, the structure of “Abc:1234” is expressed as “Aaa#1111”.

### Sentence

Sentence based features intend to reflect general characteristics of the sentence where the target token is present. Features are provided to reflect the number of tokens present on each sentence. Considering an average number of 25 tokens per sentence, we decided to generate the following seven clusters: 1) less than 15 tokens; 2) between 15 and 20 tokens; 3) between 20 and 25 tokens; 4) between 25 and 30 tokens; 5) between 30 and 35 tokens; 6) between 35 and 40 tokens; and 7) more than 40 tokens.

### Concepts

These features reflect information regarding the concept annotations previously provided, such as gene and protein names. Four different types of concept-based features are generated:

● Tags: a tag is provided when the token is part of a concept name, such as “Concept = Protein”;

● Names: the names of the concepts in the sentence are also added as features. When the concept name contains more than one token, it is concatenated with “_”. For instance, considering the protein in Figure 2, the feature “CONCEPT_NAME = interferon_regulatory_factor_4” is added to all the tokens in the sentence;

● Heads: a feature is added to reflect the head token of the concept name. For instance, considering the protein name “interferon regulatory factor 4” (Figure 2), the feature “CONCEPT_PROTEIN_HEAD = interferon” is added to all the tokens in the sentence;

● Counting: a feature is added with the number of annotations per concept type in the sentence. For instance, if the sentence containing the token has two genes and one chemical annotation, the features “NUM_PROTEIN = 2” and “NUM_CHEMICAL = 1” are added to each token in the sentence.

### External resources

Further optimization can be achieved by adding biomedical knowledge to the feature set. To provide this knowledge, dictionaries of specific domain terms and trigger words are matched in the text and the resulting tags are used as features. Thus, the tokens that are part of a matched term contain a feature that reflects such information. For instance, if a dictionary of gene expression triggers is provided, and the token “coexpressed” is matched, the feature “Trigger = Gene_expression” is added to the token.

### Dependency parsing

The previous features provide a local analysis of the sentence. To complement these with information about relations between the tokens of a sentence, we use features derived from dependency parsing. First, we consider modifier features that could indicate the presence of a trigger word. This is done by adding as features of each token, the lemmas corresponding to each of the following: verbs for which the token acts as subject; verbs for which the token acts as object; nouns for which the token acts as modifier; and the modifiers of that token.

Features to reflect input and output dependencies are also added, considering inherent dependency, lemma, POS and chunk tags. For instance, regarding the sentence of Figure 2 and the token “regulation”, the following features are added:

● Input dependencies:

○ “IN_DEP_LABEL = NMOD”;

○ “IN_DEP_LEMMA = in”;

○ “IN_DEP_POS = PP”;

○ “IN_DEP_CHUNK = PP”;

● Output dependencies:

○ “OUT_DEP_LABEL = OBJ”;

○ “OUT_DEP_LEMMA = −”;

○ “OUT_DEP_POS = HYPH”;

○ “OUT_DEP_CHUNK = O”.

By analyzing the dependency parse graph, we can find the shortest paths between two different tokens, by applying the Dijkstra's algorithm [27]. Since biomedical events and their triggers rely on entity names, it should be informative to extract features to reflect the relation between each token and the closest entity name. For instance, as illustrated in Figure 2, the shortest path between the token “regulation” and the closest entity “interferon regulatory factor 4”, is “regulation-of-expression-4”. Specific to shortest paths, we provide a feature to reflect the shortest distance between the current token and the closest entity name. Again, considering the token “regulation” on Figure 2, it should contain the feature “SPDistance = 3”, which is the number of hops between the token and the closest entity.

For both dependency and shortest paths, the following features are added (examples are based on the tokens “regulation” and “4” of Figure 2):

● Edge path: path of edge labels between two tokens (e.g., “NMOD-PMOD-NMOD”);

● Edge type: reflect the type of path based on its size and first edge label (e.g., “NMOD_3”);

● Vertex path: path of features of tokens (vertexes) between two tokens (e.g., “regulation-of-expression-4”, considering lemmas as features);

● Edge n-grams: n-grams of edge labels between two tokens (e.g., “NMOD_PMOD” and “PMOD_NMOD”, considering 2-grams);

● Vertex n-grams: n-grams of features of tokens (vertexes) between two tokens (e.g., “regulation_of”, “of_expression” and “expression_4”, considering 2-grams and lemmas as features).

### Context

Higher-level relations between tokens and extracted features can be established through windows or conjunctions of features, reflecting the local context of each token. Conjunctions consist of creating new features by grouping together features of the surrounding tokens. For instance, considering the token “regulatory” in the sentence of Figure 2 and a {−1,1} window, the new conjunction feature “interferon@-1_&_factor@1” is created. The windows {−3,-1}, {−2,-1}, {−1,0}, {−1,1} and {0,1} are used with lemmas and POS tags, which have been shown to provide positive outcomes on biomedical concept recognition [23]. On the other hand, the application of windows consists of adding selected features from surrounding tokens, selected following two different interpretations of neighborhood: local and dependency. Local windows add features of preceding and succeeding tokens as features of the current token. The offset positions considered are the same as those applied for conjunctions, but using token, lemma, POS and chunk features. Regarding dependency windows, the tokens are selected following the linguistic dependencies provided by dependency parsing. For instance, considering the token “regulation” in the sentence of Figure 2 and a maximum of 1 hop, features of the tokens “of”, “-” and “in” would be used. In the end, we consider a maximum of 3 hops and take the lemma, POS and chunk features of each token in that neighborhood.

### Optimization algorithm

Since triggers for different event types have different characteristics in terms of textual context and linguistic construction, we believe that training a CRF model focused on each event type will deliver improved results in terms of accuracy and speed. Thus, the optimization algorithm aims to find the feature set and model parameters that better reflect the characteristics of each event type. The proposed method considers the following optimization targets:

● Feature set: choose the features that better reflect the linguistic characteristics of the triggers for a particular event type.

● Context: choose the technique that provides a better representation of local context.

● Model orders: choose the model order that better fits the linguistic characteristics of the triggers.

● N-grams sizes: find the n-grams size that better reflects the common sub-structures of the triggers

● Maximum hops on dependency parsing: choose the maximum number of hops used to extract dependency parsing-based features.

● Feature extracted from vertex on dependency parsing associated features: during the construction of dependency parsing-based features, optimize the information used from each vertex.

Table 1 presents the pseudo-code and processing pipeline of the optimization algorithm, assuming the following notation:

● *D*: data set:

○ *D*_*T*_: train data set;

○ *D*_*D*_: development data set;

● *M*: model;

● *MC*: model configurations;

● *T*: trigger types;

● *F*: feature set;

● *O*: model orders;

● *N*: n-grams;

● *FN*: features that use n-grams;

● *C*: contexts;

● *H*: dependency hops;

● *FH*: features that use dependency hops;

● *V*: vertex feature type;

● *FV*: features that use vertex type.

**Table 1:** Pseudo-code of the optimization algorithm

**Optimization**_**(*D*, T, *F*, *O*, *C*, *N*, *H*, *V*)**_				
1)	randomly split dataset *D* into train *D*_*T*_ and development *D*_*D*_ datasets			
2)	for each trigger type *T*_*i *_*ϵ T*			
	a) for each feature type *F*_*j *_*ϵ F*			
	i) activate feature *F*_*j*_ on model configuration *MC*_*i*_			
	ii) call TrainModels with *D*_*T*_, *D*_*D*_, *MC*_*i*_ and *O*			
	iii) if no improvement, deactivate feature *F*_*j*_ on model configuration *MC*_*i*_			
	b) for each context type *C*_*j *_*ϵ C*			
	i) activate context *C*_*j*_ on model configuration *MC*_*i*_			
	ii) call TrainModels with *D*_*T*_, *D*_*D*_, *MC*_*i*_ and *O*			
	c) store best performing context on model configuration *MC*_*i*_			
	d) for each feature with n-grams *FN*_*j *_*ϵ FN*			
	i) for each n-grams *N*_*k *_*ϵ N*			
	(1) activate n-gram *N*_*k*_ for feature *FN*_*j*_ on model configuration *MC*_*i*_			
	(2) call TrainModels with *D*_*T*_, *D*_*D*_, *MC*_*i*_ and *O*			
	ii) store best performing n-gram of feature *FN*_*j*_ on model configuration *MC*_*i*_			
	e) for each feature with dependency hops *FH*_*j *_*ϵ FH*			
	i) for each dependency hop *H*_*k *_*ϵ H*			
	(1) activate hop *H*_*k*_ for feature *FH*_*j*_ on model configuration *MC*_*i*_			
	(2) call TrainModels with *D*_*T*_, *D*_*D*_, *MC*_*i*_ and *O*			
	ii) store best performing n-gram of feature *FN*_*j*_ on model configuration *MC*_*i*_			
	f) for each feature with vertex feature type *FV*_*j *_*ϵ FV*			
	i) for each vertex feature type *V*_*k *_*ϵ V*			
	(1) activate vertex type *V*_*k*_ for feature *FV*_*j*_ on model configuration *MC*_*i*_			
	(2) call TrainModels with *D*_*T*_, *D*_*D*_, *MC*_*i*_ and *O*			
	ii) store best performing vertex type of feature *FV*_*j*_ on model configuration *MC*_*i*_			
3) Return MC				
**Train models** (*D*_*T*_, *D*_*D*_, *MC*_*i*_, *O*)				
1) for each *O*_*j *_*ϵ O*				
	a) train model *M* on dataset *D*_*T*_ using *MC*_*i*_			
	b) get performance of model *M* on dataset *D*_*D*_			
	c) store performance and model order if better			
2) return better performance and order				

Optimization algorithm arguments (_*T*_, _*F*_, _*O*_, _*N*_, _*C*_, _*H*_) are entirely configurable, allowing users to easily customize optimization goals, workflow and complexity. Additionally, default values are assumed unless others are provided. For instance, considering the array of contexts [None, Window, Conjunctions], *None* is considered the default value until further optimization is performed. The same approach is applied for n-gram sizes, maximum hops and vertex features. By analyzing the “TrainModels” method, which is used on every training task, we can see that a model is trained for each order, considering the various model orders *o* during the entire optimization process. Regarding the “Optimization” method, which considers each trigger type from _*T*_, it starts by iteratively choosing the best feature set from _*F*_, followed by the best local context technique selection from _*C*_. Afterwards, alternative optimizations are performed, choosing the best n-grams size for each feature in _*FN*_, selecting the best maximum number of hops for each dependency parsing feature in _*FH*_, and choosing the best vertex information for each vertex-based dependency parsing feature in _*FV*_. During this process, if a feature is not used in the feature set, it is skipped from further optimization. When the optimization process finishes, the final model configurations are obtained, with optimized feature set and parameters for each event type. In the end, the final model for each event type is trained using the obtained model configuration and the complete train data set, and stored.

### Annotation

In order to annotate hundreds of documents using multiple ML models with different feature sets, we have to avoid generating the complete feature set for each ML model. Thus, a strategy must be applied to extract all the required features at once and filter them per model. To achieve this, a model configuration that results from the union of all model configurations is built and used to extract all the required features. Afterwards, the features are filtered per model, respecting the optimized requirements of each model, and the corpus is annotated using these models. By applying this strategy we considerably reduce the complexity of annotating a corpus with multiple ML models, since extracting some complex features may take considerable amounts of time and computational resources.

### Post-processing

Post-processing tasks can be performed to further optimize and/or filter the identified event triggers. Three different approaches are implemented, based on:

● Parentheses: if the number of parentheses (round, square and curly) on each annotation is an odd number, the annotation is removed since it clearly indicates a mistake by the ML model;

● Concepts: the trigger annotation is removed if the sentence does not contain any concept annotation;

### Output

The output can be generated in various formats, namely JSON, XML and A1, the default, which is the official format for the BioNLP challenges. A sample output is shown in the bottom of Figure 2, composed of a unique identifier, the event type, start and end character positions, and the chunk of text.

## Results

This section presents the performance results achieved on a manually annotated corpus. A detailed comparison with other existing approaches is performed, and the annotation and optimization speeds are analyzed.

### Corpus

To provide a fair comparison of the achieved performance results in terms of event trigger recognition, we used an annotated corpus with manually annotated triggers and events. As stated before, the BioNLP challenges have highly promoted the extraction of biomedical events, especially in the recognition of gene and protein-based events. Moreover, since the training and development data sets provided in the first two BioNLP GENIA challenges (2009 and 2011) are similar, we decided to use the corpus of the BioNLP 2009 GENIA shared task [28] since more results were available for comparison. This corpus contains manual event annotations for nine biomedical events, categorized into three different groups:

● Simple events: gene expression, transcription, protein catabolism, phosphorylation and localization.

● Binding events: binding.

● Regulation events: regulation, positive regulation and negative regulation.

The corpus contains training and development parts, which we used to train the ML models and compare final performance results, respectively. Table 2 presents a detailed analysis of the corpus parts and the provided manual annotations, namely proteins, events and triggers.

**Table 2:** Statistics of the training and development data sets of the BioNLP 2009 GENIA shared task: number of abstracts, sentences, annotated proteins, events and triggers

	**Train**	**Development**
Abstracts	800	150
Sentences	≈7449	≈1450
Proteins	9300	2080
Events	8615	1795
Triggers	7041	1476

### Experiment

**Figure 4:**
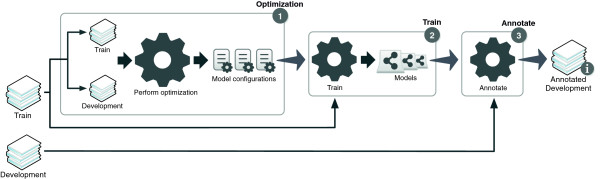
Illustration of the workflow applied to perform optimization, train the final models and annotate the development corpus.

Figure 4 illustrates the workflow applied to perform optimization (1), train the final models (2), and annotate the development set (3) for evaluation and comparison. Here we split the training dataset into two parts in order to train and optimize the system. Moreover, the original development dataset is used as the test dataset. The optimization algorithm was executed with the following input arguments:

● Triggers (_*T*_): [Gene_expression, Transcription, Protein_catabolism, Phosphorylation, Localization, Binding, Regulation, Positive_regulation, Negative_regulation];

● Feature set (_*F*_): all features;

● Orders (*o*): [1-3];

● Contexts (*c*): [None, Window, Dependency Window, Conjunctions];

● N-grams (_*N*_): [2-4], [2,3], [3,4];

● Hops (_*H*_): [2,3];

● Vertex types (*v*): [Lemma, Token, POS, Chunk].

Additional file 1: Table S1 presents the model configurations obtained after running the optimization algorithm. As can be observed, each event type requires a different feature set, reflecting the heterogeneous linguistic and context characteristics. As expected, simple events require simpler feature sets in comparison to regulatory events, whose feature sets include more token-based, concept-based and syntactic information, in order to properly model the higher complexity associated with their phrasal structure and linguistic contexts. Moreover, we also observed that the reduced amount of examples for some event types, namely protein catabolism, phosphorylation and localization, is also reflected in the complexity of the feature set, since fewer features are required to model the lower heterogeneity present in these fewer cases. By contrast, the feature sets to recognize gene expression, transcription and binding events require a considerable amount of context and dependency parsing information. Overall, higher order CRF models are preferred, with seven out of nine event trigger types requiring CRFs of order three. This reflects a strong dependency on accurate sequence prediction, which we believe is directly associated with the inherent linguistic complexity of event descriptions. The low impact of local context features was unexpected, since they provide an important contribution in the case of biomedical concept recognition. However, we believe that this reduced contribution is a consequence of the deeper context description provided by dependency parsing features. Finally, we can observe that shortest path features have a much more relevant contribution than dependency path features, showing that, as expected, establishing a relation with concept names in the sentence is fundamental in the recognition of event trigger words.

### Evaluation metrics

Since more than 90% of trigger expressions are a single token, we believe that there is no need to apply fuzzy matching techniques for evaluation. Thus, only exact matching is applied, accepting an annotation as correct only if both left and right sides match. Standard evaluation metrics are used to analyze and compare the achieved results: Precision (the ability of a system to present only relevant items); Recall (the ability of a system to present all relevant items); and F-measure (the harmonic mean of precision and recall). These measures are formulated as follows:

$$P=\frac{\mathrm{TP}}{\mathrm{TP}+\mathrm{FP}},\;R=\frac{\mathrm{TP}}{\mathrm{TP}+\mathrm{FN}},\;F1=2\cdot \frac{P\times R}{P+R},$$

where *TP* is the amount of true positives, *FP* the number of false positives and *FN* the amount of false negatives. Note that the presented results are micro-averaged, meaning that a general matrix of *TP*, *FP* and *FN* values is built from all documents to obtain final precision, recall and F-measure scores.

### Results

**Figure 5:**
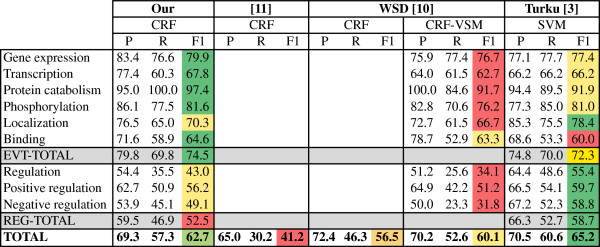
Detailed performance results achieved by the proposed automatic approach compared with existing state-of-the-art systems.

Figure 5 details the results of the proposed event trigger recognition method in the development set of the BioNLP 2009 GENIA shared task, and compares this with other existing systems. The data show that our approach achieves state-of-the-art results, with an F-measure of 74.5 on simple events and 52.5 on regulatory events. Overall, it achieves an F-measure of 62.7. Comparing with other existing systems, it achieves the best results on simple events, outperforming other solutions on gene expression, transcription, protein catabolism, phosphorylation and binding event triggers. Overall, our approach presents the second best results, due to the significant performance differences for regulation and negative regulation events, on which it is considerably outperformed by the best performing system. Nonetheless, the presented results are comparable to the best ones previously reported for this task and show the positive contribution of a simple automatic optimization approach.

Regarding the application of CRFs, our solution considerably outperforms previous systems, with an overall difference of more than 6 points of F-measure. This shows that CRFs are able to provide positive results in the recognition of event trigger words.

### Speed

In order to analyze the applicability of our approach in large-scale problems, it is important to analyze the annotation processing speeds. There are various factors that add complexity to our system, namely dependency parsing, feature extraction and annotation with multiple ML models. However, the applied annotation algorithm together with multithreaded processing reduces the processing times significantly. Considering the complete processing pipeline presented on Figure 2 and the complexity associated with the previously obtained model configurations, the 1450 sentences of the development set of the BioNLP 2009 shared task were annotated in 40 seconds (excluding the time required to load processing models) on a machine with 8 processing cores @ 2.67 GHz and 16GB of RAM and using four processing threads. Thus, our system is able to process more than 36 sentences/second, corresponding to almost 4 abstracts/second. We believe that these results present a positive contribution, considering the inherent complexity and obtained performance results.

Regarding the optimization algorithm, this requires significant computational resources and may take a considerable amount of time, depending on the optimization algorithm configuration. In our case, which considered a high variety of complex features and parameters, the optimization process took almost 24 hours to find the best model configurations for nine event types. Thus, on average, about 2.6 hours were necessary to find the best model configuration for each event type.

## Discussion

The solution presented in this article was built thinking on flexibility and configurability. Its architecture allows easy inclusion of new functionalities and modules, enabling easy development of new feature extraction algorithms and its integration in complex event extraction solutions. Additionally, considering the extracted linguistic information and its structured storage and access, and the amount of already implemented ML features, we believe that our solution is also a good starting point for the development of event extraction systems. Moreover, the approach and research presented in this article provides a new perspective of the linguistic and context complexity associated with each event trigger, providing a better perception of the associated requirements. This information is useful for the implementation of new event and trigger extraction solutions.

Regarding the optimization algorithm, it was developed to be completely configurable, allowing developers to easily specify the feature set, n-grams sizes, model orders and maximum dependency parsing hops. Such flexibility facilitates adapting the tool to new corpora, different domains and event triggers. Typically, the development of NER or trigger recognition solutions is performed by manually selecting the feature set and parameters that provide the best results, which is a very demanding and time-consuming task. The presented approach is able to automatically find high-performance models in just a few hours, which we believe will save researchers’ time. Since the optimization process only has to be executed once for any particular corpus, we consider the presented optimization times acceptable, in comparison with the time required to manually perform a similar process. Moreover, considering the variety of possible biomedical events, as can be seen from the new tasks emerging in the BioNLP challenges [3,4], we can argue that the presented automatic optimization approach is an added value.

As previously shown, the automatic approach proposed here presents state-of-the-art results in the recognition of nine heterogeneous event triggers, outperforming existing solutions on simple event triggers. However, we believe there is still a margin to improve results on regulation events, which can be accomplished through the integration of new features for improved context description. By comparing the achieved performance results, we also showed that CRFs are able to perform as well as SVMs in the recognition of event triggers, considerably outperforming previous CRF-based approaches through appropriate context definition features. Additionally, our approach also presents positive annotation processing speeds, enabling its application in large-scale problems, such as annotating the entire MEDLINE.

## Conclusions

This article presents TrigNER, a new tool for biomedical event trigger recognition, taking advantage of a flexible and configurable optimization algorithm that allows the tool to adapt itself to corpora with different events and domains while maintaining high-performance results. It takes advantage of CRFs and feature sets optimized for the linguistic and context characteristics of each event type. The application of this automatic optimization algorithm delivered state-of-the-art performance results on the BioNLP 2009 shared task corpus with a total F-measure of 62.7 and outperformed existing solutions on various event trigger types, namely gene expression, transcription, protein catabolism, phosphorylation and binding.

We believe that TrigNER represents a valuable contribution to the biomedical text mining community, by providing simplified event trigger recognition. Researchers can use it to replace or complement non-state-of-the-art dictionary-based approaches, taking advantage of a complex and high-performance solution and applying it as a simple and routine task, therefore leveraging their time to optimize and improve event argument extraction algorithms. Thus, this research work contributes to an improved, grounded and faster development of biomedical event extraction solutions, leading to the identification of hidden relations and facilitating knowledge discovery.

## Competing interests

The authors declare that they have no competing interests.

## Authors’ contributions

DC and QCB participated in the design and implementation of the tool and drafted the manuscript. SM and JLO conceived the study, participated in its coordination and helped to draft the manuscript. All authors read and approved the final manuscript.

## Supplementary Material

Additional file 1: Table S1 Detailed description of the model configurations obtained after running the automatic optimization algorithm. Click here for file
