# Supplementary material for: TrigNER: automatically optimized biomedical event trigger recognition on scientific documents
Source: Source Code Biol Med. 2014 Jan 8;9:1. doi: 10.1186/1751-0473-9-1 (PMC3896761; doi:10.1186/1751-0473-9-1)
Supplement: Additional file 1: Table S1 — Detailed description of the model configurations obtained after running the automatic optimization algorithm. [file 1751-0473-9-1-S1.docx]

Additional file 1

Table S1: Detailed description of the model configurations obtained after running the automatic optimization algorithm in the training data. Configurations presented as “Lemma, [2,3,4], 3”, indicate the applicability of [2,3,4] n-grams to combine lemmas of each vertex until a maximum number of 3 dependency hops.
